# Nitrate Promotes Germination Under Inhibition by NaCl or High Concentration of Glucose

**DOI:** 10.3390/plants9060707

**Published:** 2020-06-02

**Authors:** Shun Ikeya, Takuya Aoyanagi, Imari Ishizuka, Ayano Takeuchi, Akiko Kozaki

**Affiliations:** 1 Department of Biological Science, Faculty of Science, Shizuoka University, 836 Ohya Suruga-ku, Shizuoka 422-8529, Japan; ike-shun0824.ngy.gpe@docomo.ne.jp (S.I.); etc-botanistnum1@docomo.ne.jp (T.A.); 2 Kato Gakuen Gyoshu High School, Numazu 410-0011, Japan; ishizumari4@gmail.com; 3 Shizuoka Gakuen High School, Shizuoka 420-0833, Japan; takeaya0325@gmail.com

**Keywords:** seed germination, *Arabidopsis*, nitrate, NaCl, glucose, abscisic acid (ABA), CYP707A2, SPATULA (SPT)

## Abstract

Seed germination, one of the most important stages in a plant’s life cycle, can be affected by abiotic stresses, such as salinity. The plant hormone abscisic acid (ABA) and high concentrations of glucose are also known to inhibit germination. In contrast, nitrate is known to stimulate germination in many plants. However, this stimulatory effect has not yet been investigated in the presence of inhibitory effects caused by abiotic stresses, ABA, and glucose. In this study, we show that nitrate can alleviate the inhibitory effects of sodium chloride (NaCl) or high concentrations of glucose on seed germination in *Arabidopsis*, while it was not able to promote germination that was inhibited by exogenous ABA and mannitol (an inducer of osmotic stress). An analysis of the gene expression involved in the regulation of germination showed that *GA20ox1,* encoding the gibberellin (GA) synthesis enzyme, *SPATULA* (*SPT*), encoding a bHLH transcription factor, and *CYP707A2,* encoding an ABA catabolic enzyme, were significantly upregulated by the addition of KNO_3_ in the presence of NaCl or glucose. Our results suggest the possibility that these genes are involved in the nitrate-mediated control of seed germination in the presence of NaCl or glucose.

## 1. Introduction

Germination is a critical stage in the life of spermatophytes that is elaborately controlled by environmental factors such as light, temperature, water, and nutrients, in addition to endogenous signals such as the balance of phytohormones, gibberellin (GA), and abscisic acid (ABA) [1,2]. The influence of environmental factors on seed germination mostly occurs through the metabolism and signaling pathways of GA and ABA [3,4], where ABA promotes and maintains dormancy, while GA promotes germination [5].

In the GA metabolism, the enzymes involved in the final step of active GA synthesis, namely the GA 20-oxidase (GA20ox), GA 3-oxidase (GA3ox), and the enzyme involved in the deactivation of GAs, namely the GA 2-oxidase (GA2ox), are considered to be important in regulating seed germination. The major GA signaling components are the DELLA proteins, which belong to the GRAS family of transcription factors. These proteins inhibit plant growth and germination by negatively regulating the GA signaling (Table S1) [6,7].

Some enzymes of the ABA metabolism, for example the 9-cis-epoxy carotenoid dioxygenase (NCED), that catalyzes the synthesis of xanthoxine in plastids, and the cytochrome p450 type CYP707As, that catalyze ABA deactivation, resulting in phaseic acid (PA) and dihydrophaseic acid (DPA), are considered to be important for the regulation of seed germination [8]. Among the ABA signaling components, the ABSCISIC ACID INSENTIVE (ABI) 3 and 5 are well known to be involved in the regulation of seed germination (Table S1) [9,10].

The regulation mechanisms of seed germination have been extensively investigated in *Arabidopsis*. *Arabidopsis* seeds require after-ripening or low temperatures to break dormancy, and light to germinate. Recent studies identified many genes that are involved in the regulation of germination by light and dormancy-breaking (by cold stratification) also to be involved in the balance of GA and ABA [6,7,11]. Light induces the breakdown of PHYTOCHROME INTERACTING FACTOR 3 -LIKE5 (PIL5 or PIF1) proteins, which suppress germination in the dark by inducing the SOMNUS *(SOM)* and MOTHER OF FT and *TFL1 (MFT)* genes [12,13]. Cold stratification decreases the expression of *SOM* and *MFT*, but increases the expression of *SPATULA* (*SPT*) genes, resulting in the breakdown of dormancy. In addition, *SPT* promotes germination by repressing *MFT* under red light conditions [13]. These genes were shown to affect the expression of other genes involved in the GA or ABA metabolism and/or signaling, either directly or indirectly (Table S1) [13].

Nitrate is known to stimulate germination in a wide variety of plant species [14,15]; therefore, it is used as an agent for seed priming [16]. The effect of nitrate on germination does not depend on nitrate reductase (NR) [14,17], indicating that nitrate itself promotes seed germination. Recent research has shown that nitrate induced the expression of *CYP707A2* gene in imbibed *Arabidopsis* seeds, and the *cyp707a2* mutant was less sensitive to nitrate during both seed development and germination [18,19]. In addition, the NIN-like protein 8 (NLP8) was found to regulate *CYP707A2* by directly binding to the *CYP707A2* promoter region required for nitrate induction. As such, the *nlp8* mutant was nonresponsive to nitrate. These results suggest that *NLP8* and its downstream *CYP707A2,* are key genes in the nitrate-regulated germination in *Arabidopsis* [19] (Table S1).

Nitric oxide (NO) is another nitrogen compound that promotes germination and induces the expression of *CYP707A2* [20,21]. Therefore, it was postulated that NO would act downstream of the nitrate signaling [22]. Recent research showed that in the presence of NO, group VII of the ethylene response factors (ERFs) is destroyed through the N-end rule pathway. Group VII ERF is involved in ABA signaling by regulating the expression and activity of ABI5 [23]. Mutants defective in the N-end rule pathway and *abi5* displayed a NO-insensitive germination [23,24], however they were sensitive to nitrate. *ABI5* expression was not altered in the *nlp8* mutant [19]. Thus, all these results indicate that NO signaling seems to be more than just a simple, linear pathway, downstream of nitrate signaling.

It is well known that germination is also affected by abiotic stresses, such as salinity, drought, and unfavorable temperature [25] although the molecular mechanism by which these stresses inhibit germination has not been completely understood. In addition to these stresses, germination is also inhibited by high concentrations of glucose (or sucrose) and ABA, as mentioned above [3,26,27].

Although it is important to know whether nitrate can stimulate germination, even in the presence of inhibitors such as abiotic stresses, ABA, and high concentrations of glucose, it has not yet been investigated. In this study, we investigated the effects of nitrate on germination affected by salt, osmotic stress (mannitol), ABA and glucose, using *Arabidopsis* seeds. We found that nitrate promoted germination under inhibition by NaCl or high concentrations of glucose, but not under exogenously applied ABA and mannitol. Gene expression analysis showed that in addition to *CYP707A2*, the expression of *GA20ox1* and *SPT* were increased by KNO_3_ in the presence of NaCl or glucose.

## 2. Materials and Methods

### 2.1. Plant Materials and Seed Germination Assays

*Arabidopsis* (Col-0) plants were grown in growth room at 23 °C under 16 h light/ 8 h dark cycle (100 μmol m^−^^2^ sec^−1^). Seeds were harvested when siliques turned dry on the plants. Seeds were stored in a dry box at 23 °C before use.

For the germination assay, seeds stored for one-two months were used. For the germination assay of freshly harvested seeds, seeds just after harvested were used.

These seeds were surface sterilized in 20% bleach for 5 min, rinsed five times with sterile water, and then plated on 0.8% agar plates. To examine the effect of NaCl and mannitol, half-strength Murashige and Skoog (1/2 MS) medium or distilled water (DW), containing 0, 102, 170, and 340 mM NaCl, or 0, 100, 200, 400, and 500 mM mannitol, respectively, were used. Since the 1/2 MS medium already contains nitrates as macronutrients, to examine the effect of these nitrogen compounds we prepared the DW+N medium, which contained the same concentration of nitrates (9 mM KNO_3_ and 10 mM NH_4_NO_3_) that are found in the 1/2 MS.

To examine the effect of KNO_3_ or NH_4_NO_3_, a medium containing 9 mM KNO_3_ and either 170 mM NaCl or 278 mM glucose (DW + KNO_3_ + NaCl or Glu), and a medium containing 10 mM NH_4_NO_3_ and either 170 mM NaCl or 278 mM glucose (DW + NH_4_NO_3_ + NaCl or Glu) were prepared. To examine the effect of NH_4_Cl, a DW + NH_4_Cl + NaCl or Glu medium that contained 10 mM NH_4_Cl and 170 mM NaCl or 278 mM glucose, respectively, were prepared. To examine the effect of KCl, a DW + KCl + NaCl or Glu medium that contained either 10 mM KCl and 170 mM NaCl or 278 mM glucose, respectively, were prepared.

Plates with seeds were first placed at 4 °C for 3 days (stratification) and then transferred to a growth chamber at 23 °C with continuous light (65 μmol m^−^^2^ sec^−1^). We used continuous light to avoid the effect of a light-dark cycle during the germination assay. Germination was scored by radicle emergence at 24, 32, 48, 56, 72, and 80 h after being transferred to 23 °C. In the experiments with glucose, germination scoring at 96 and 104 h was included. In the experiments with freshly harvested seeds, the germination was scored until 200 h (with additional scoring at 120, 128, 144, 152, 168, 176, 192, and 200 h). Each plate contained 50 seeds, and three plates were used for each experiment. The data on the final germination of each experiment were analyzed using an ANOVA followed by Tukey’s test.

### 2.2. RNA Extraction

Approximately 30 mg of seeds were sown on sterile filters in Petri dishes (60 × 15 mm), containing DW or 10 mM KNO_3_ solution (DW + KNO_3_) containing 170 mM NaCl, 278 mM glucose, 5 μM ABA, or 500 mM mannitol. Plates with seeds were first placed at 4 °C for 3 days and then transferred to a growth chamber at 23 °C with continuous light. After 6 or 24 h of incubation in a growth chamber, seeds were collected and stored at −80 °C for RNA extraction.

Total RNA from seeds was isolated using the ISOGEN II (Nippon Gene, Tokyo, Japan) and Fruit-mate (TAKARA, Ohtsu, Japan) reagents. cDNA was synthesized (from 0.5 μg RNA template) using the ReverTra Ace Kit (TOYOBO, Osaka, Japan) according to the manufacturer’s instructions.

### 2.3. Quantitative Reverse Transcription Polymerase Chain Reaction (qRT-PCR)

Real-time PCR amplification of cDNAs was conducted using a LightCycler 480 (Roche Diagnostics, Rotkreuz, Switzerland) in a 384-well PCR plate. The reactions were carried out in 10 μL reaction volumes, containing 5 μL of FastStart Essential DNA Green Master (Roche Diagnostics) with 0.2 μM of the forward and reverse primers and 1 μL cDNA (10-fold dilution). The primer sets used for real-time PCR are shown in Table S2. The primers used in this experiment were designed by the Universal ProbeLibrary Assay Design Center (Roche Diagnostics). The *ELONGATION FACTOR1 αA4* (*EF1αA4*) gene was used for signal normalization in the real-time PCR.

Relative expression levels were calculated using the 2^−^^ΔΔCT^ method [28]. All PCR reactions were performed at least five times, out of which at least three sets of consistent data were used for the analyses. In order to validate the reliability of data, we compared the amplification efficiencies of the target and reference genes for all PCR reactions, and examined the dissociation curves for all PCR products. Data were statistically analyzed by a Student’s t test.

### 2.4. Semi-Quantitative RT-PCR

These PCR reactions were performed with the Emeraldamp PCR master mix (Takara) using the following program: 98 °C for 2 min; 30–35 cycles of 15 s at 98 °C, 30 s at 55 °C, and 0.5–2 min at 72 °C; then hold at 72 °C for 5 min. The *ACTIN2/8* gene was used as a reference gene. The primer sets used for the expression analysis are shown in Table S2.

### 2.5. Statistical Analysis

Statistical analysises were performed using the EZR software [29] which is based on R commander.

## 3. Results

### 3.1. Germination in the Presence of NaCl or Mannitol

**Figure 1:**
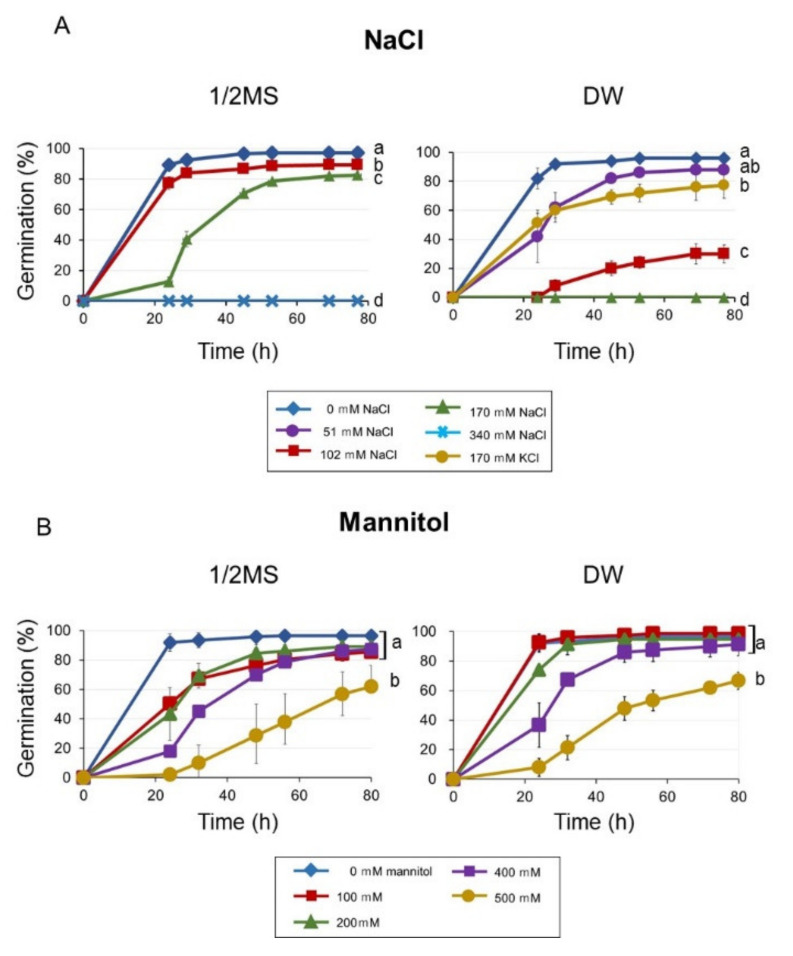
Seed germination on 1/2 MS or distilled water (DW), in the presence of several concentrations of NaCl (**A**) and mannitol (**B**) Seeds were stratified for three days before incubation at 23 °C. Germination was scored after transferred to 23 °C. Data are means ± standard deviation (SD) of three replicates. Each replicate contained 50 seeds. Different letters indicate significant differences in germination at the final time point (ANOVA and Tukey’s test, *p* < 0.05).

We examined the inhibitory effect of NaCl and mannitol (inducer of osmotic stress) on the germination of *Arabidopsis* seeds, and found that germination was reduced on 1/2 MS containing 170 mM (1%) NaCl, and was completely inhibited on 340 mM NaCl (Figure 1A). Interestingly, germination was already reduced at 102 mM NaCl and 170 mM NaCl could inhibit germination fully in DW. The same concentration of KCl (170 mM) delayed germination only slightly, indicating that the inhibition was caused mainly by sodium and not chloride. Mannitol delayed germination in a concentration-dependent manner, and the germination was significantly reduced by 500 mM mannitol in both 1/2 MS and DW. The effect of mannitol on germination was similar between treatment using 1/2 MS and DW as the medium (Figure 1B).

**Figure 2:**
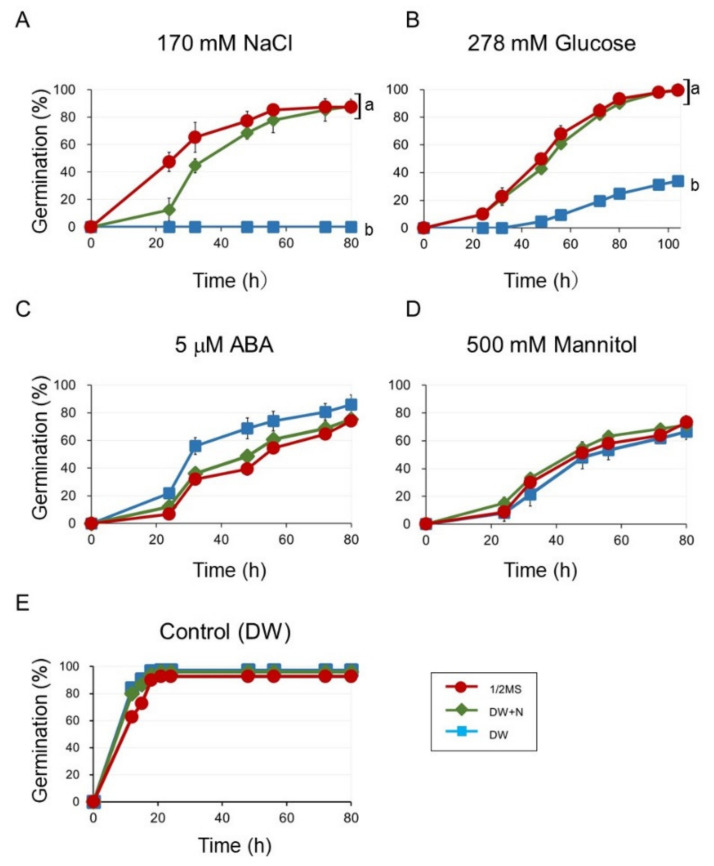
Seed germination on 1/2 MS, nitrogen components of 1/2 MS (DW+N), and DW, in the presence of (**A**) 170 mM NaCl, (**B**) 278 mM glucose, (**C**) 5 μM ABA, and (**D**) 500 mM mannitol. (**E**) controls (no inhibitory compounds) Seeds were stratified for three days before incubation at 23 °C. Germination was scored after transferred to 23 °C. Data are means ± SD of three replicates with each replicate containing 50 seeds. Different letters indicate significant differences in germination at the final time point (ANOVA and Tukey’s test, *p* < 0.05).

Since the germination-promoting effects of nitrogen compounds, such as nitrate and nitrogen oxide (NO) are known [14,19,22,30], we speculated that nitrogen (N) components in 1/2 MS medium would promote germination in the presence of 170 mM NaCl. Therefore, we examined the germination on both 1/2 MS and DW in the presence of N compounds (DW + N: 9 mM KNO_3_ and 10 mM NH_4_NO_3_) containing 170 mM NaCl (Figure 2). The germination on DW + N containing 170 mM NaCl (DW + N + NaCl) was almost the same as on 1/2 MS containing 170 mM NaCl (1/2 MS + NaCl) (Figure 2A). The results suggested that the reason why the germination on 1/2 MS + NaCl was better than that on DW + NaCl was mainly due to the presence of N compounds in the 1/2 MS medium. We also examined the germination on DW + N containing 500 mM mannitol (DW + N + Man), which was almost the same as that on DW containing 500 mM mannitol (DW + Man) (Figure 2D).

The differences observed in the effect of N compounds on the germination inhibited by NaCl or mannitol prompted us to analyze other germination inhibitors, such as ABA and high concentrations of glucose. Interestingly, germination in DW containing 5 μM ABA (DW + ABA) was inhibited to a similar extent as that on 1/2 MS containing 5 μM ABA (1/2 MS + ABA) and DW+N containing 5 μM ABA (DW + N + ABA) (Figure 2C), whereas germinations on 1/2 MS containing 278 mM (5%) glucose (1/2 MS + Glu) and DW + N containing 278 mM glucose (DW + N + Glu) were much higher than that on DW containing 278 mM glucose (DW + Glu) (Figure 2B).

Higher germination in the presence of NaCl or glucose on 1/2 MS or DW + N were observed when the germination assay was carried out using freshly harvested seeds without stratification (Figure S1).

### 3.2. Nitrate Promotes Germination Inhibited by NaCl or High Concentrations of Glucose

**Figure 3:**
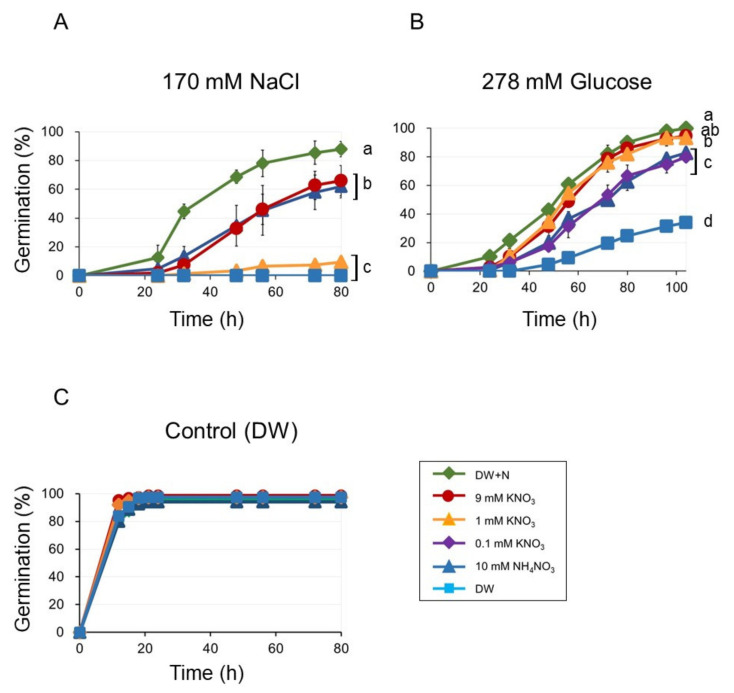
Seed germination on KNO_3_ or NH_4_NO_3_ medium (DW, DW + N, 9 mM KNO_3_, 10 mM NH_4_NO_3_, 1 mM KNO_3_ and 0.1 mM KNO_3_) in the presence of inhibitory compounds (**A**) 170 mM NaCl, (**B**) 278 mM glucose, and (**C**) controls (no inhibitory compounds). Seeds were stratified for three days before incubation at 23 °C. Germination was scored after transferred to 23 °C. Data are means ± SD of three replicates. Each replicate contained 50 seeds. Different letters indicate significant differences in germination the final time point (ANOVA and Tukey’s test, *p* < 0.05).

As we used a mixture of 9 mM KNO_3_ and 10 mM NH_4_NO_3_ as nitrates in the media, we had to examine the effect of each nitrogen compound on germination individually. Our experimental setup consisted of germinations inhibited by NaCl or Glu (DW + NaCl or DW + Glu) in the presence of either 9 mM KNO_3_ (DW + KNO_3_ + NaCl or DW + KNO_3_ + Glu) or 10 mM NH_4_NO_3_ (DW + NH_4_KO_3_ + NaCl or DW + NH_4_NO_3_ + Glu). For comparison, we also included germination with DW + N + NaCl and DW + N + Glu. The germination for DW + KNO_3_ + NaCl and DW + NH_4_NO_3_ + NaCl were similar but slower than that on DW + N + NaCl (Figure 3A). However, when we reduced KNO_3_ concentration to 1 mM, the germination was also reduced (Figure 3A).

Furthermore, we decided to analyze the effect of ammonium salt (NH_4_Cl) instead of NH_4_NO_3_, as the latter contains both NH_4_^+^ and NO_3_^−^. The germination rate in DW + NaCl containing 10 mM NH_4_Cl (DW + NH_4_Cl + NaCl) was better than in DW + 1 mM KNO_3_ + NaCl, but worse than in DW + KNO_3_ + NaCl (Figure S2A). Since KNO_3_ also contains potassium (K^+^), we examined the effect of potassium using KCl. The germination rate in DW + NaCl containing 10 mM KCl (DW + KCl + NaCl) was similar to that of DW + NH_4_Cl + NaCl, indicating that both NH_4_Cl and KCl could improve germination in the presence of NaCl, although the effects were weaker than for KNO_3_ (Figure S2A).

The germination rates were similar in DW + KNO_3_+ Glu and DW + 1 mM KNO_3_ + Glu (Figure 3B). Moreover, germination in DW + Glu was similarly improved by 0.1 mM KNO_3_ and 10 mM NH_4_NO_3_ (Figure 3B). Germination in DW + Glu containing 10 mM NH_4_Cl (DW + NH_4_Cl+Glu) or 10 mM KCl (DW + KCl + Glu) was only slightly lower than in DW + KNO_3_ + Glu (Figure S2B). Thus, a much lower KNO_3_ concentration was already effective at enhancing the germination inhibited by glucose than what we observed for NaCl.

In the control experiments (without any inhibitory compounds), no significant differences in germination were observed under both KNO_3_ and NH_4_NO_3_ (Figure 3C).

Furthermore, we decided to analyze the effect of ammonium salt (NH_4_Cl) instead of NH_4_NO_3_, as the latter contains both NH_4_^+^ and NO_3_^−^. The germination rate in DW + NaCl containing 10 mM NH_4_Cl (DW + NH_4_Cl + NaCl) was better than in DW + 1 mM KNO_3_ + NaCl, but worse than in DW + KNO_3_ + NaCl (Figure S2A). Since KNO_3_ also contains potassium (K^+^), we examined the effect of potassium using KCl. The germination rate in DW + NaCl containing 10 mM KCl (DW + KCl + NaCl) was similar to that of DW + NH_4_Cl + NaCl, indicating that both NH_4_Cl and KCl could improve germination in the presence of NaCl, although the effects were weaker than for KNO_3_ (Figure S2A).

The germination rates were similar in DW + KNO_3_ + Glu and DW + 1 mM KNO_3_ + Glu (Figure 3B). Moreover, germination in DW + Glu was similarly improved by 0.1 mM KNO_3_ and 10 mM NH_4_NO_3_ (Figure 3B). Germination in DW + Glu containing 10 mM NH_4_Cl (DW + NH_4_Cl+Glu) or 10 mM KCl (DW + KCl + Glu) was only slightly lower than in DW + KNO_3_ + Glu (Figure S2B). Thus, a much lower KNO_3_ concentration was already effective at enhancing the germination inhibited by glucose than what we observed for NaCl.

In the control experiments (without any inhibitory compounds), no significant differences in germination were observed under both KNO_3_ and NH_4_NO_3_ (Figure 3C).

### 3.3. Gene Expression in Seeds Imbibed with NaCl, Glucose, ABA, and Mannitol

**Figure 4:**
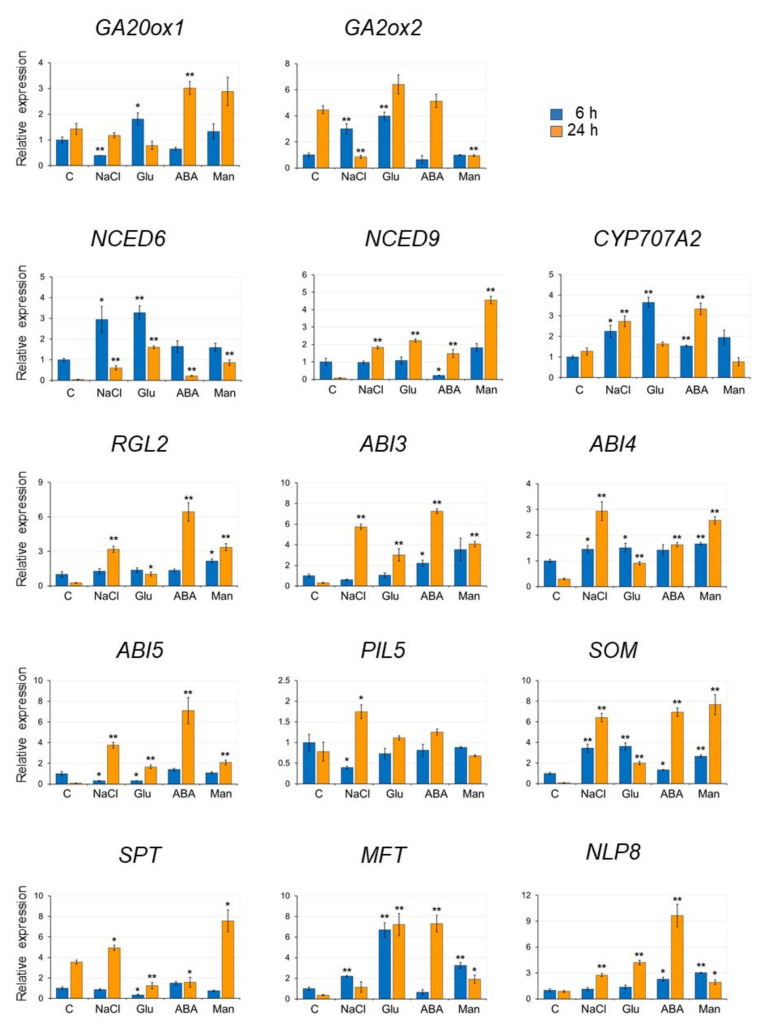
Expression of genes involved in the regulation of seed germination in the presence of NaCl, glucose, ABA, and mannitol. Seeds were imbibed in the presence of 170 mM NaCl, 278 mM glucose (Glu), 5 μM ABA, and 500 mM mannitol (Man) for 6 h and 24 h. C: control (no inhibitory compounds). Gene expression was quantified by quantitative RT-PCR (qRT-PCR) and presented as relative to the levels in the control seeds (imbibed for 6 h). The expression levels were normalized against the expression of *ELONGATION FACTOR1 αA4* (*EF1αA4*). Data are presented as the mean ± SD of three replicates. Asterisks indicate a significant difference between control and each treatment at *p* < 0.01 (^**^) and *p* < 0.05 (^*^). (Student’s t-test).

Next, we analyzed the gene expression in seeds imbibed in the presence of several inhibitory compounds, namely, 170 mM NaCl, 278 mM glucose, 5 μM ABA, and 500 mM mannitol by qRT-PCR (Figure 4). The length of the imbibing period (6 and 24 h) was chosen to detect both rapid and slower changes in gene expression, as many of the genes could be detected within 24 h of imbibition, while genes that are rapidly induced by nitrate are detected within 6 h [31].

The selected genes are involved in the regulation of seed germination, specifically genes encoding enzymes involved in GA synthesis (*GA20 ox1*), GA catabolism (*GA2ox1*), ABA synthesis (*NCED6* and *NCED9*), ABA catabolism (*CYP707A2*), DELLA (*RGL2*), factors involved in ABA signaling (*ABI3*, *ABI4*, and *ABI5*) and seed germination by light and temperature (*PIL5*, *SOM*, *SPT*, and *MFT*), plus *NLP8,* which was identified as an important factor in promoting *CYP707A2* expression by the addition of nitrate (Table S1) [19].

Figure 4 shows the relative expression of each gene in seeds imbibed with NaCl, glucose, ABA, and mannitol for 6 or 24 h, compared with their expression in seeds imbibed for 6 h in DW. The expression pattern of each gene differed depending on the added inhibitory compounds. Since the expression of *NCED6*, *NCED9*, *RGL2*, *ABI3*, *ABI4*, *ABI5*, *SOM*, and *MFT* decreased after 24 h imbibition in DW, the relative expression of these genes in seeds imbibed in the presence of the inhibitory compounds was much higher than in seeds imbibed in DW. Overall, we found these inhibitory compounds to induce the expression of genes that repress germination or that are involved in ABA synthesis and ABA signaling.

### 3.4. Gene Expression in Seeds Imbibed With or Without KNO_3_ in the Presence of NaCl, Glucose, ABA, or Mannitol

We compared the gene expression in seeds imbibed with or without 10 mM KNO_3_ in the presence of NaCl, glucose, ABA, or mannitol, by semi-quantitative RT-PCR (Figure S3). We used KNO_3_ because of its widespread use in studies that examined the effect of nitrates on germination [14,18,19]. In addition to the genes shown on Figure 4, we also analyzed other genes by semi-quantitative RT-PCR: namely, genes for GA synthesis (*GA3ox1* and *GA3ox2*), *DELLAs* (*GAI*, *RGA*, *RGL1*, and *RGL3*), and *EXPANSINs* (*EXP1* and *EXP2*). The semi-quantitative RT-PCR analysis showed that the expression of several genes was altered by the addition of nitrate (Figure S3). The change in gene expression by the addition of KNO_3_ was different, depending on the added inhibitory compounds, so we could not find any common effect of KNO_3_ on gene expression. Since we could not detect significant changes in the expression of *GA3oxes*, *DELLAs,* and *EXPANSINs* by semi-quantitative analysis in the presence or absence of KNO_3_, we decided to examine the genes from Figure 4 by qRT-PCR.

**Figure 5:**
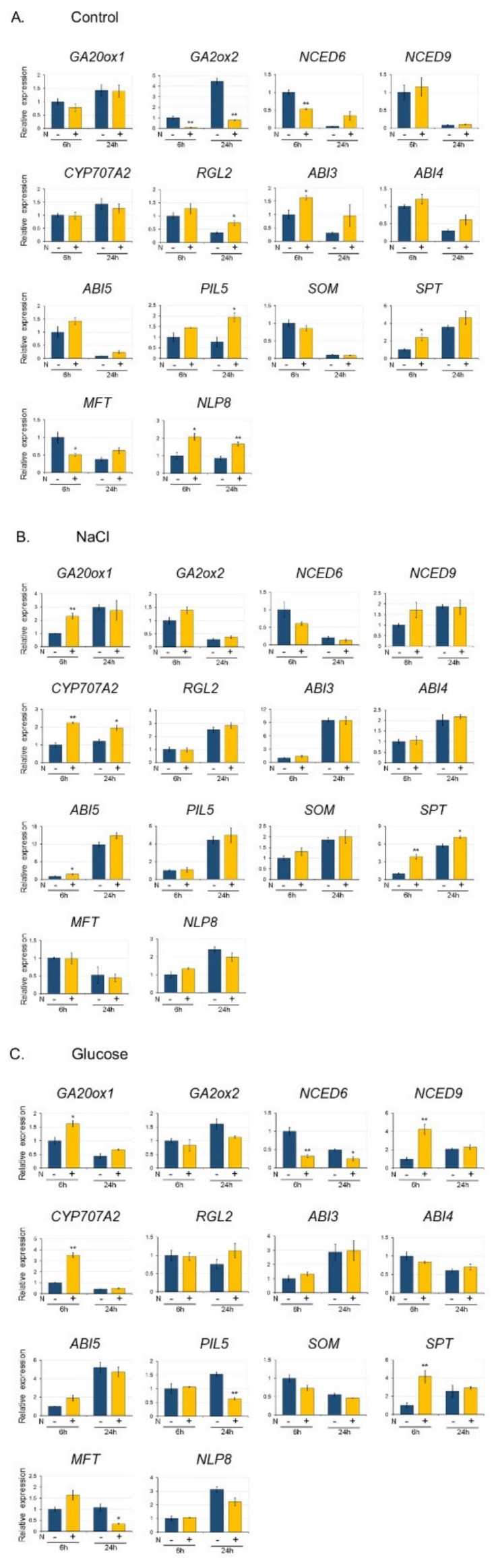
Expression of genes involved in the regulation of seed germination in the presence (+) or absence (−) of 10 mM KNO_3_. Seeds were imbibed in (**A**) control: no additional compounds, (**B**) in the presence of 170 mM NaCl, (**C**) in the presence of 278 mM glucose, for 6 and 24 h, respectively. Gene expression was quantified by qRT-PCR and values are presented as relative to the control seeds (imbibed for 6 h). The expression levels were normalized against the expression of *ELONGATION FACTOR1 αA4* (*EF1αA4*). Data are presented as the mean ± SD for three replicates. Asterisks indicate a significant difference between the absence (−) and the presence (+) of KNO_3_ at *p* < 0.01 (^**^) and *p* < 0.05 (^*^) (Student’s t-test).

Because the addition of KNO_3_ increased the germination in the presence of 170 mM NaCl or 278 mM Glu, we compared the gene expression in seeds imbibed with or without KNO_3_ in the presence of NaCl or glucose (Figure 5).

In the absence of NaCl or Glu, *GA2ox2* expression was significantly reduced by KNO_3_ in both 6 h and 24 h imbibition (Figure 5A). Although *NLP8* expression was increased by the addition of KNO_3_, the expression of *CYP707A2* did not change after either 6 or 24 h of imbibition. *SPT* expression however, increased, while *MFT* expression was decreased by KNO_3_ after 6 h of imbibition (Figure 5A).

In the presence of 170 mM NaCl, the expression of *GA20ox1* was higher after 6 h of imbibition with KNO_3_, while the expression of *GA2ox2* did not change. Interestingly, the expression of *CYP707A2* was increased after both 6 and 24 h imbibition with KNO_3_, although the expression of *NLP8* did not change. The expression of *SPT* was increased in both 6 h and 24 h imbibition with KNO_3_, while the expression of *MFT* did not change (Figure 5B).

In the presence of 278 mM Glu, the expression of *GA20ox1*, *NCED9*, and *CYP707A2* increased after 6 h of imbibition with KNO_3_, while the expression of *NCED6* decreased following both 6 and 24 h of imbibition with KNO_3_. Moreover, *SPT* expression also increased after 6 h of imbibition with KNO_3_, while that of *MFT* decreased after 24 h of imbibition (Figure 5C).

## 4. Discussion

Nitrate has been shown to be one of the signals that relieve seed dormancy in many species, including *Arabidopsis* [14,15]. However, the effect of nitrate on germinations affected by abiotic stresses, ABA, and glucose has not been investigated. Our study showed that nitrate was able to enhance germinations that were inhibited by NaCl and high concentrations of glucose, but was unsuccessful in promoting germinations inhibited by exogenous ABA and mannitol (Figure 2).

It is well known that salt affects seed germination [32,33,34], however, the mechanisms by which salt inhibits seed germination remain largely unknown. Salinity stress by NaCl consists of a primarily osmotic stress plus the toxic effect of Na^+^. Since our results indicated that the germinations inhibited by osmotic stress were not improved by the addition of nitrate (Figure 2), it seems that the toxicity of Na^+^ could have been mitigated by nitrate. Ethylene signaling has been reported to modulate salt response, including germination inhibition [35] and recent research revealed the roles of ethylene signaling in salt stress. Yu et al. (2016) showed that salt treatment caused the CONSTITUTIVE PHOTOMORPHOGENESIS 1 (COP1) protein to be retained in the cytosol and inhibited the interaction between COP1 and LONG HYPOCOTYL 5 (HY5) [36]. COP1, a ring finger E3 ligase, is translocated to the nucleus in the dark and is responsible for the proteasome-mediated degradation of photomorphogenesis-promoting factors, such as HY5 [37,38]. HY5 has been reported to mediate ABA response through *ABI5* during seed germination and early seedling growth [35]. Therefore, HY5 activates ABA signaling in the presence of salt stress to inhibit germination. In contrast, ethylene enhances the localization of COP1 to the nucleus to promote germination [36]. In addition, Li et al. (2015) reported that NO managed to alleviate germination inhibition induced by salt stress, by enhancing ethylene signaling [39]. In many plants, NO can be produced from nitrate by nitrate reductase (NR) [31,40]. Therefore, it could be possible that the effect of nitrate on germination inhibited by NaCl is attributed to the effect of NO, through the activation of ethylene signaling. However, our expression analysis showed that the expression of *ABI5* was not significantly different in the presence or the absence of nitrate (Figure 5), suggesting that nitrate did not attenuate ABA signaling through ABI5 in our experimental setup. However, further research is required to elucidate whether the effect of nitrate is related to NO and/or ethylene signaling.

In general, the expression of all examined genes was affected by the addition of NaCl (Figure 5B), but only the expression of *GA20ox1*, *CYP707A2*, and *SPT* was upregulated by the addition of KNO_3_ in the presence of NaCl. *CYP707A2* has been reported to be a key gene for the enhancement of seed germination by nitrate in *Arabidopsis* [8,18], so the observed increase in the expression of *CYP707A2* could have contributed to the improvement of germination in the presence of NaCl.

By modulating gene expression and influencing a variety of processes such as germination, early seedling development, flowering, or senescence, glucose plays an important regulatory role as a central signaling molecule [40].

High concentrations of glucose are known to delay germination in several plants [26,27,41], but the mechanisms by which this happens have not been completely clarified. Exogenously applied high concentrations of glucose during germination lead to enhancement of ABA biosynthesis [42] and the repression of genes associated with ABA catabolism [27]. It has been reported that the delay of germination by glucose is not caused by increased cellular ABA concentration, but rather by the fact that glucose appears to slow down the decline of endogenous ABA. Moreover, the glucose-induced delay in germination seems to be independent from hexokinase (HXK) [26,42,43].

Our expression analysis showed that genes of the ABA biosynthesis (*NCED6* and *NCED9*) and ABA signaling (*ABI3*, *4*, and *5*) were increased by the addition of 278 mM glucose (Figure 4). *CYP707A2*, however, involved in ABA degradation, was also increased by the addition of glucose (Figure 4). This result contradicts other reports according to which the expression of *CYP707A2* was reduced by the addition of glucose [27,43]. It is not clear why these expression data were different. In rice seeds the expressions of *OsABA8ox2* and *OsABA8ox3* (rice *CYP707As* orthologues) were decreased by 1% glucose, but their expression increased to a level that was similar in control (0% glucose) under higher concentrations (5%) of glucose [27]. The glucose concentration used in our experiment was 5%, which is higher than the one used by Zhu et al. (1% and 3%) in *Arabidopsis* [43]. Hence, it is possible that the response of *CYP707A2* expression to glucose might be concentration-dependent.

With the addition of KNO_3_, the expression of *CYP707A2* was higher than in control at 6 h, while the expression of *NCED6* was lower at 6 and 24 h in the presence of 278 mM glucose. In contrast, *NCED9* expression was higher than in control at 6 h, and dropped to a similar level at 24 h (Figure 5C). These results indicated that the expression of genes involved both in ABA biosynthesis and catabolism were affected by KNO_3_. In contrast, the expression of *RGL2* and genes for ABA signal transduction were not changed by KNO_3_. Interestingly, *SPT* expression was significantly reduced by the addition of glucose (Figure 4), and it was increased by KNO_3_ application (Figure 5C). On the other hand, *MFT* expression was increased by the addition of glucose (Figure 4), but it was suppressed by KNO_3_ (Figure 5C). Therefore, the assumption that *MFT* and *SPT* would be directly involved in the regulation of germination by glucose and KNO_3_ needs further clarification.

The expression of *GA20ox1*, *CYP707A2*, and *SPT* were increased by KNO_3_ both in the presence of NaCl or glucose. The increase of *CYP707A2* and *GA20ox1* by KNO_3_ may partly explain the reason why KNO_3_ enhanced germination in the presence of NaCl or glucose. However, it is not yet clear how increased *SPT* expression could contribute to enhanced germination in the presence of NaCl or glucose.

The function of *SPT* in the physiology of germination are complicated. For example, *SPT* induces the expression of *ABI5* and *RGL3,* while it represses that of *MFT*, *ABI4,* and *RGA* in freshly matured *Arabidopsis* seeds. As a result, *SPT* promotes dormancy in *Columbia* ecotype, but represses it in the *Landsberg erecta* ecotype [44]. However, *SPT* promotes germination in imbibed seeds, by repressing *MFT* under red light conditions [13]. It will be interesting to elucidate whether *SPT* is involved in the enhancement of germination by KNO_3_ in the presence of NaCl or glucose.

The fact that KNO_3_ did not alleviate the effects of inhibition by exogenously supplied ABA on the germination (Figure 2) was an unexpected finding, although the expression of *CYP707A2* was induced by the addition of KNO_3_ both in our current and previous studies [18,19]. The addition of KNO_3_ triggered an increase in the expression of *CYP707A2* in the presence of 5μM ABA (Figure 4), however it is possible that this increase was not enough to reduce the exogenous ABA level. However, KNO_3_ application did not enhance germination even when this was inhibited by a much lower concentration of ABA (1 μM) (Figure S4). The mapping of genes involved in the regulation of germination that code for enzymes involved in ABA synthesis and ABA degradation revealed overlapping expression patterns (during germination) in the root cap, epidermis, and vascular cells of the radicle in *Arabidopsis* embryos. On the other hand, the expression of ABA response-genes has been mainly localized to the outer cell layers of the embryo radicle, principally the root cap and epidermis [45]. These results indicate that, while endogenously-produced ABA is degraded efficiently, the degradation of exogenously-applied ABA may not be efficient, despite an increase in the *CYP702A2* expression.

Finally, our germination analysis showed that both NH_4_Cl and KCl could alleviate the NaCl—or glucose-induced inhibition of germination, although they were less effective than KNO_3_ (Figure S2). Ammonium salts and other nitrogenous compounds have been reported to promote germination in some plants, but the mechanisms behind this have not been clarified [46]. These nitrogenous compounds yield NO under strong oxidation, and the produced NO might promote germination [30]. However, it is not clear whether the effect of NH_4_Cl on germination in our experiment is through the production of NO. Although KCl is sometimes used for seed priming [30], the mechanism by which KCl stimulates germination has also not been clarified. Thus, the elucidation of how these compounds can improve germination under inhibition by NaCl or glucose is of great importance.

Further research will be needed, with a comprehensive temporal and spatial gene expression analysis, to elucidate how the inhibition of germination under unfavorable conditions (by high salinity and glucose) are alleviated by nitrate.

## Supplementary Materials

The following are available online at https://www.mdpi.com/2223-7747/9/6/707/s1. Figure S1: Seed germination on 1/2 MS, nitrogen components of 1/2 MS (DW+N), and DW in the presence of (**A**) 170 mM NaCl, (**B**) 278 mM glucose, and (**C**) no additional compounds (control)., Figure S2: The effect of KCl and NH_4_Cl on seed germination in the presence of (**A**) 170 mM NaCl and (**B**) 278 mM Glucose., Figure S3: Expression of genes involved in the regulation of seed germination in the presence in the presence (+N) or absence (−N) of 10 mM KNO_3_., Figure. S4. Seed germination on 1/2 MS, nitrogen components of 1/2 MS (DW + N), and DW in the presence of 1 μM ABA. Table S1: List of genes used in this study., Table S2: Primers used in this study.
