# Supplementary material for: Nitrate Promotes Germination Under Inhibition by NaCl or High Concentration of Glucose
Source: Plants (Basel). 2020 Jun 2;9(6):707. doi: 10.3390/plants9060707 (PMC7355496; doi:10.3390/plants9060707)
Supplement: Supplementary file 1 [file plants-09-00707-s001.pdf]

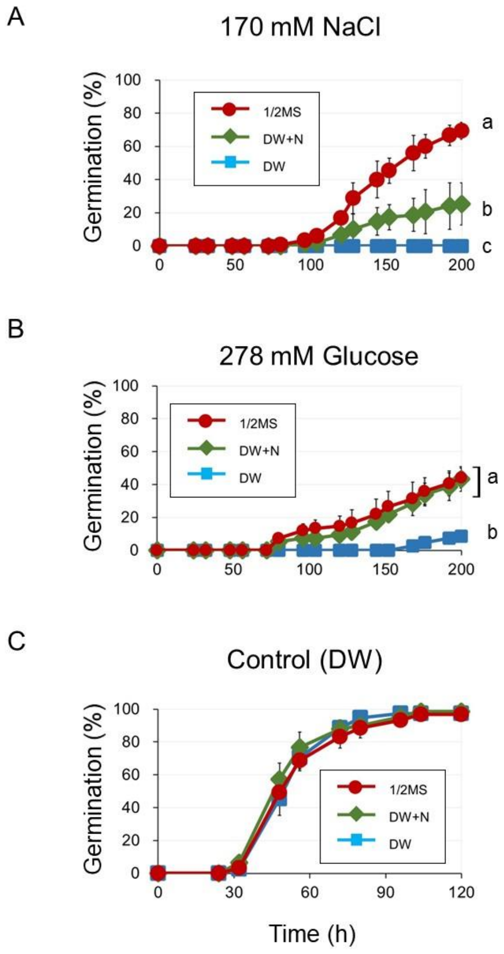

Figure S1. Seed germination on 1/2 MS, nitrogen components of 1/2 MS (DW+N), and DW in the presence of (**A**) 170 mM NaCl, (**B**) 278 mM glucose, and (**C**) no additional compounds (control). Freshly harvested seeds were incubated at 23 °C without stratification. Data are means ± SD for three replicates. Each replicate containing 50 seeds. Different letters indicate significant differences in germination at the final time point. (ANOVA and Tukey's test, $p < 0.05$).

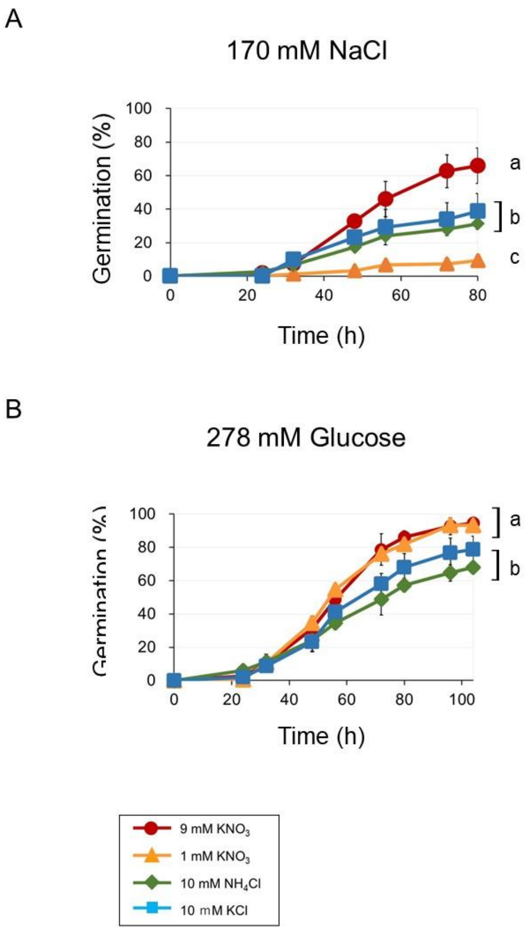

Figure S2. The effect of KCl and NH_4Cl on seed germination in the presence of (**A**) 170 mM NaCl and (**B**) 278 mM Glucose. Seeds were stratified for three days before incubation at 23 °C. Germination was scored after transferred to 23 °C. Data are means $\pm$ SD for three replicates. Each replicate containing 50 seeds. Different letters indicate significant differences in germination at the final time point. (ANOVA and Tukey's test, $p < 0.05$).

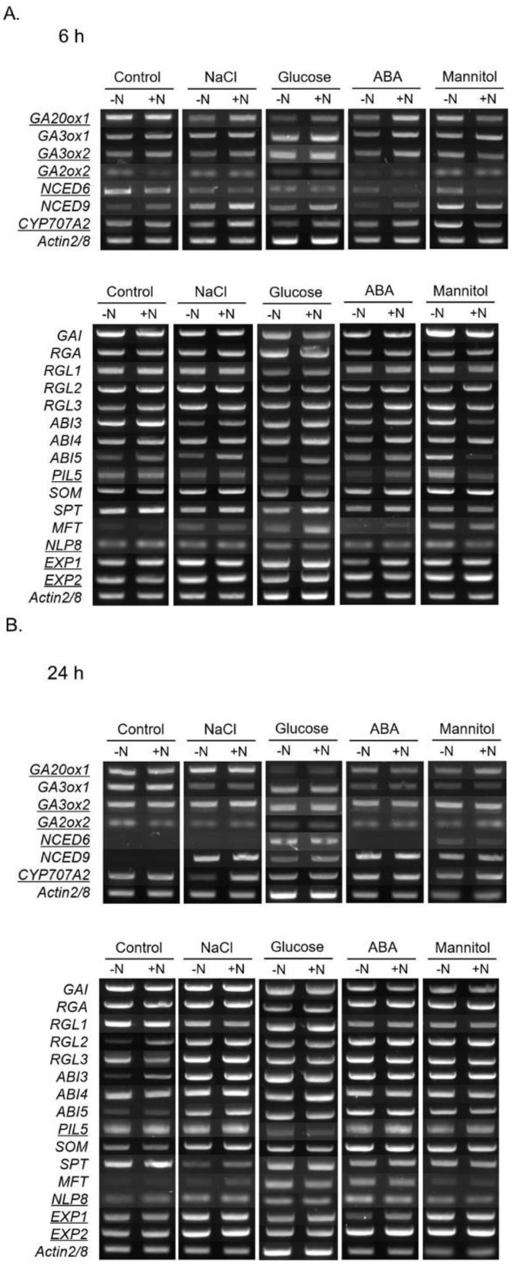

Figure S3. Expression of genes involved in the regulation of seed germination in the presence in the presence (+N) or absence (-N) of 10 mM KNO_3. Seeds were imbibed in the presence of 170 mM NaCl, 278 mM Glucose, 5 mM ABA, and 500 mM Mannitol for (**A**) 6 and (**B**) 24 h. The gene expression was analyzed by semi-quantitative RT-PCR. The Actin 2/8 gene was used as control. The gene amplification reactions were carried out for 30 PCR cycles or 35 PCR cycles (underlined genes).

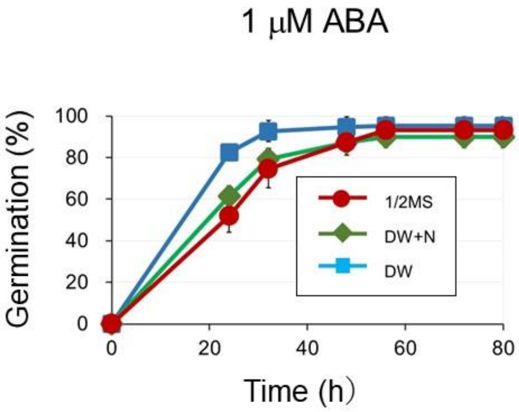

Figure S4. Seed germination on 1/2 MS, nitrogen components of 1/2 MS (DW + N), and DW in the presence of 1 μM ABA. Seeds were stratified for two days before incubation at 23 °C. Data are means ± SD for three replicates. Each replicate containing 50 seeds.

Table S1. List of genes used in this study.

Gene Name	Protein	Key role	References
GA20ox1 (*At4g25420*) *GA20-OXIDASE 1*	gibberellin 20-oxidase	GA biosynthesis	[47,48]
GA3ox1 (*At1g15550*) *GA3-OXIDASE 1*	Gibberellin 3-b-dioxygenase	GA biosynthesis	[49] [50]
GA3ox2 (AT1G80340) GA3-OXIDASE 2	Gibberellin 3-b-dioxygenase	GA biosynthesis	[49]
GA2ox2 (AT1G30040) GA2-OXIDASE 2	Gibberellin 2-b-dioxygenase	GA cabatolism	[51,52]
NCED6 (AT3G24220) NINE-CIS-EPOXYCAROTENOID DIOXYGENASE 6	9-cis-epoxycarotenoid dioxygenase	ABA biosynthesis	[53]
NCED9 (AT1G78390) NINE-CIS-EPOXYCAROTENOID DIOXYGENASE 9	9-cis-epoxycarotenoid dioxygenase	ABA biosynthesis	[53]
CYP707A2 (AT2G29090) ABA 8′-HYDROXYLASE CYTOCHROME P450-2	Abscisic acid 8'-hydroxylase	ABA degradation	[54,55]
GAI (AT1G14920) GA INSENSITIVE	DELLA transcription factor	GA signaling	[56]
RGA (AT2G01570) REPRESSOR OF GA1-3	DELLA transcription factor	GA signaling	[57]
RGL1 (AT1G66350) RGA-LIKE 1	DELLA transcription factor	GA signaling	[58]
RGL2 (AT3G03450) RGA-LIKE 2	DELLA transcription factor	GA signaling	[59]
RGL3 (AT5G17490) RGA-LIKE 3	DELLA transcription factor	GA signaling	[60]
ABI3 (AT3G24650) ABA INSENSITIVE 3	b-ZIP transcription factor	ABA signaling	[60]
ABI4 (AT2G40220) ABA INSENSITIVE 4	AP2 transcription factor	ABA signaling	[61]
ABI5 (AT2G36270) ABA INSENSITIVE 5	b-ZIP transcription factor	ABA signaling	[60]
PIL5 (AT2G20180) PHYTOCHROME INTERACTING FACTOR 3-LIKE 5	PIF1, bHLH transcription factor	light signaling	[62,63]
SOM (AT1G03790) SOMNUS	zinc finger CCCH-type transcription factor	seed germination	[12]
SPT (AT4G36930) SPATULA	bHLH transcription factor	floral organogenesis.seed germination	[64]
MFT (AT1G18100) MOTHER OF FT AND TFL1	phosphatidylethanolamine-binding protein	seed dormancy, germination	[65]
NLP8 (AT2G43500) NIN-LIKE PROTEIN 8	NIN-like transcription factor	nitrate signaling	[19]
EXP1 (AT1G69530) EXPANSIN 1	expansin	loosening and extension of plant cell walls	[66]
EXP2 (AT5G05290) EXPANSIN 2	expansin	loosening and extension of plant cell walls	[67]

Table S2. Primers used in this study.

**Semi-quantitative RT-PCR (GA/ABA metabolism)**	
GA20ox1 F	ATGGCCGTAAGTTTCGTAAC
GA20ox1 R	TTAGATGGGTTTGGTGAGCC
GA3ox1 F	ATGCCTGCTATGTTAACAG
GA3ox1 R	TCATTCTTCTCTGTGATTTC
GA3ox2 F	ATGAGTTCAACGTTGAGCG
GA3ox2 R	TTAATTTCTAATAATGGAAAG
GA2ox2 F	ATGGTGGTTTTGCCACAGCC
GA2ox2 R new	TCATACAAGGGTTTTATGATTG
NCED6 F	ATGCAACACTCTCTTCGTTC
NCED6 R	TCAGAAAACTTGTTCCTTCAAC
NCED9 3rd ATG- F	ATGGCTTCTACTACCTTACT
NCED9 R	CTAACACAAAGCTTGCTTCG
CYP707A2 F	ATGCAAATCTCATCTTCATC
CYP707A2 R	TTAAATCGGGGTTACTCTTATTG
**Semi-quantitative RT-PCR (GA/ABA signaling)**	
Y GAI EcoRI F	GGAATTCATGAAGAGAGATCATC
Y GAI BamHI R	GGGATCCCTAATTGGTGGAGAGTTTCC
RGA1 XbaI F	GTCTAGAATGAAGAGAGATCATCACCAATTCCAAG
RGA1 BamHI R	GGGATCCTCAGTACGCCGCCGTCGAGAGTTTCCAAGC
Y RGL1 BamHI F	GGGATCCCGATGAAGAGAGAGCACAACCAC
Y RGL1 SalI R	GGTCGACTTATTCCACACGATTGATTC
Y RGL2 SalI F	GGTCGACCGATGAAGAGAGGATACGGAGAAAC
Y RGL2 PstI R	GCTGCAGTCAGGCGAGTTTCCACGCCG
Y RGL3 SalI F	GGTCGACGATGAAACGAAGCCATCAAG
Y RGL3 PstI R	GCTGCAGCTACCGCCGCAACTCCGCCG
ABI3 XbaI F	GTCTAGAATGAAAAGCTTGCATGTGGC
ABI3 BamHI R	GGGATCCTCATTTAACAGTTTGAGAAG
ABI4 XbaI F for effector	GTCTAGAATGGACCCTTTAGCTTCCC
ABI4 KpnI R for effector	GGGTACCTTAATAGAATTCCCCCAAG
ABI5 BamHI F	GGGATCCATGGTAACTAGAGAAACGAAG
ABI5 KpnI R	GGGTACCTTAGAGTGGACAACTCGGGTTC
SPATULA KpnI F	CGGTACCATGATATCACAGAGAGAAG
SPATULA EcoRI R	GGAATTCTCAAGTAATTCGATCTTTTAG
Y MFT EcoRI F	GGAATTCATGGCGGCTTCTGTTGATCC
Y MFT SalI R	GGTCGACCTAGCGTCTGCGTGAAGCAG
PIL5 XbaI F	GTCTAGAATGCATCATTTTGTCCCTG
PIL5 KpnI R	GGGTACCTTAACCTGTTGTGTGGTTTC
SOMNUS BamHI F	GGGATCCATGGATGTCGTTTGTACGG
SOMNUS KpnI R	GGGTACCTCAAGTCAAGAGATCATTG
EXP1 F	ATGGCTCTTGTCACCTTCTTG
EXP1 R new	TCAAGCACTCGAAGCACCAC
EXP2 F	ATGAATCTTACAGAATATTC
EXP2 R	CTAAAATTGTCCGCCTTC
NLP8_1500bp_F	TTTGAGAACTGTTTCAGAAG
NLP8_2000bp_R	TCGCCAGTTGCTGAGTCGAAC
Actin2/8 F	CTGGTGATGGTGTGTCTCAC
Actin2/8 R	GACCTTAATCTTCATGCTGC
**Quantitative RT-PCR**	
Real-time GA20ox1(Roche) F	CATGGGTTTCAGCCATTTG
Real-time GA20ox1(Roche) R	CTCTAAAGTAGTCCCGTTTTACGC
Real-time GA2ox2(Roche) F	CTCCTCCTCAATGCTAATCCTC
Real-time GA2ox2(Roche) R	CTCCTCCACCGACTCACG
Real-time NCED6(Roche) F	TAGCAATAGCCGATCCTTGG
Real-time NCED6(Roche) R	GCCGTTTTGTATATCTACCTTCG
Real-time NCED9(Roche) F	AAAGTATATTTACGGAGAGGGGAAAT
Real-time NCED9(Roche) R	CGTCACCGGAAGGTAGAAAC
Real-time CYP707A2(Roche) F	GCTTCTCAAGTACTTACACGACCA
Real-time CYP707A2(Roche) R	TGTCGAATGCTGAATTGCTC
Real-time PIL5 (Roche) F	AGCGAGGAAACAAAACAAGC

Real-time PIL5 (Roche) R	TCATTGATCCTATCTCTCCGTTTT
Real-time SOM (Roche) F	GAGAACGTTTCCGGTGTCTC
Real-time SOM (Roche) R	CAAGCAACATGGATTCTTCG
Real-time SPT (Roche) F	GCAGAGCTGCTGAAGTTCATAAT
Real-time SPT (Roche) R	TTTGAATTAGGGATGAGACTTTGTAA
Real-time MFT F (Xi et al. 2010)	CGAGCCGAACATGAGAGAAT
Real-time MFT R (Xi et al. 2010)	AAGTATCTCTTTTCCTCTTGAGGG
Real-time ABI3 (Roche) F	GCTGGCTCAGCTTCTGCTAT
Real-time ABI3 (Roche) R	AAATTCTTTTCTGGTTTCCATCC
Real-time ABI4 (Roche) F	CCGCTTCTTCTCCTTCCAC
Real-time ABI4 (Roche) R	GAGGGAGGAGAGGTCTTAGGG
Real-time ABI5 (Roche) F	TGGAGAGAAGACAGAGGAGGA
Real-time ABI5 (Roche) R	GTTCAGCTTCCAATTCCACTG
Real-time RGL2 (Roche) F	CTTTCTGCGTTTCCAAAGGA
Real-time RGL2 (Roche) R	TCGGATCCTCTTGCTGCTA
Real-time NLP8 (Roche) F	AGGCACTTTGGAGCAGGA
Real-time NLP8 (Roche) R	GCTCACATTTTTCTCTGTAGTGCT
Real-time EF1aA4 (Roche) F	CTTGGTGTCAAGCAGATGATTT
Real-time EF1aA4 (Roche) R	CGTACCTAGCCTTGGAGTATTTG

References

47. Rieu, I.; Ruiz-Rivero, O.; Fernandez-Garcia, N.; Griffiths, J.; Powers, S.J.; Gong, F.; Linhartova, T.; Eriksson, S.; Nilsson, O.; Thomas, S.G., et al. The gibberellin biosynthetic genes AtGA20ox1 and AtGA20ox2 act, partially redundantly, to promote growth and development throughout the Arabidopsis life cycle. Plant J 2008, 53, 488-504, doi:10.1111/j.1365-313X.2007.03356.x.
48. Plackett, A.R.G.; Powers, S.J.; Fernandez-Garcia, N.; Urbanova, T.; Takebayashi, Y.; Seo, M.; Jikumaru, Y.; Benlloch, R.; Nilsson, O.; Ruiz-Rivero, O., et al. Analysis of the developmental roles of the Arabidopsis gibberellin 20-oxidases demonstrates that GA20ox1, -2, and -3 are the dominant paralogs. Plant Cell 2012, 24, 941-960, doi:10.1105/tpc.111.095109
49. Yamaguchi, S.; Smith, M.W.; Brown, R.G.; Kamiya, Y.; Sun, T. Phytochrome regulation and differential expression of gibberellin 3beta-hydroxylase genes in germinating Arabidopsis seeds. Plant Cell 1998, 10, 2115-2126, doi:10.1105/tpc.10.12.2115.
50. Yamauchi, Y.; Ogawa, M.; Kuwahara, A.; Hanada, A.; Kamiya, Y.; Yamaguchi, S. Activation of gibberellin biosynthesis and response pathways by low temperature during imbibition of Arabidopsis thaliana seeds. Plant Cell 2004, 16, 367-378, doi:10.1105/tpc.018143.
51. Thomas, S.G.; Phillips, A.L.; Hedden, P. Molecular cloning and functional expression of gibberellin 2-oxidases, multifunctional enzymes involved in gibberellin deactivation. Proc Natl Acad Sci U S A 1999, 96, 4698-4703, doi:10.1073/pnas.96.8.4698.
52. Ogawa, M.; Hanada, A.; Yamauchi, Y.; Kuwahara, A.; Kamiya, Y.; Yamaguchi, S. Gibberellin Biosynthesis and Response during Arabidopsis Seed Germination. Plant Cell 2003, 15, 1591, doi:10.1105/tpc.011650.
53. Tan, B.C.; Joseph, L.M.; Deng, W.T.; Liu, L.; Li, Q.B.; Cline, K.; McCarty, D.R. Molecular characterization of the Arabidopsis 9-cis epoxycarotenoid dioxygenase gene family. Plant J 2003, 35, 44-56, doi:10.1046/j.1365-313x.2003.01786.x.
54. Kushiro, T.; Okamoto, M.; Nakabayashi, K.; Yamagishi, K.; Kitamura, S.; Asami, T.; Hirai, N.; Koshiba, T.; Kamiya, Y.; Nambara, E. The Arabidopsis cytochrome P450 CYP707A encodes ABA 8'-hydroxylases: key enzymes in ABA catabolism. EMBO J 2004, 23, 1647-1656, doi:10.1038/sj.emboj.7600121.
55. Okamoto, M.; Kuwahara A Fau - Seo, M.; Seo M Fau - Kushiro, T.; Kushiro T Fau - Asami, T.; Asami T Fau - Hirai, N.; Hirai N Fau - Kamiya, Y.; Kamiya Y Fau - Koshiba, T.; Koshiba T Fau - Nambara, E.; Nambara,

E. CYP707A1 and CYP707A2, which encode abscisic acid 8'-hydroxylases, are indispensable for proper control of seed dormancy and germination in Arabidopsis.

56. Peng, J.; Carol, P.; Richards, D.E.; King, K.E.; Cowling, R.J.; Murphy, G.P.; Harberd, N.P. The Arabidopsis GAI gene defines a signaling pathway that negatively regulates gibberellin responses. Genes & development 1997, 11, 3194-3205, doi:10.1101/gad.11.23.3194.
57. Silverstone, A.L.; Ciampaglio, C.N.; Sun, T. The Arabidopsis RGA gene encodes a transcriptional regulator repressing the gibberellin signal transduction pathway. Plant Cell 1998, 10, 155-169, doi:10.1105/tpc.10.2.155.
58. Wen, C.K.; Chang, C. Arabidopsis RGL1 encodes a negative regulator of gibberellin responses. Plant Cell 2002, 14, 87-100, doi:10.1105/tpc.010325.
59. Lee, S.; Cheng, H.; King, K.E.; Wang, W.; He, Y.; Hussain, A.; Lo, J.; Harberd, N.P.; Peng, J. Gibberellin regulates Arabidopsis seed germination via RGL2, a GAI/RGA-like gene whose expression is up-regulated following imbibition. Genes & development 2002, 16, 646-658, doi:10.1101/gad.969002.
60. Giraudat, J.; Hauge, B.M.; Valon, C.; Smalle, J.; Parcy, F.; Goodman, H.M. Isolation of the Arabidopsis ABI3 gene by positional cloning. Plant Cell 1992, 4, 1251-1261, doi:10.1105/tpc.4.10.1251.
61. Söderman, E.M.; Brocard, I.M.; Lynch, T.J.; Finkelstein, R.R. Regulation and function of the Arabidopsis ABA-insensitive4 gene in seed and abscisic acid response signaling networks. Plant Physiol 2000, 124, 1752-1765, doi:10.1104/pp.124.4.1752.
62. Oh, E.; Yamaguchi, S.; Hu, J.; Yusuke, J.; Jung, B.; Paik, I.; Lee, H.S.; Sun, T.P.; Kamiya, Y.; Choi, G. PIL5, a phytochrome-interacting bHLH protein, regulates gibberellin responsiveness by binding directly to the GAI and RGA promoters in Arabidopsis seeds. Plant Cell 2007, 19, 1192-1208, doi:10.1105/tpc.107.050153.
63. Oh, E.; Kim, J.; Park, E.; Kim, J.I.; Kang, C.; Choi, G. PIL5, a phytochrome-interacting basic helix-loop-helix protein, is a key negative regulator of seed germination in Arabidopsis thaliana. Plant Cell 2004, 16, 3045-3058, doi:10.1105/tpc.104.025163.
64. Heisler, M.G.; Atkinson, A.; Bylstra, Y.H.; Walsh, R.; Smyth, D.R. SPATULA, a gene that controls development of carpel margin tissues in Arabidopsis, encodes a bHLH protein. Development (Cambridge, England) 2001, 128, 1089-1098.
65. Xi, W.; Liu, C.; Hou, X.; Yu, H. MOTHER OF FT AND TFL1 regulates seed germination through a negative feedback loop modulating ABA signaling in Arabidopsis. Plant Cell 2010, 22, 1733-1748, doi:10.1105/tpc.109.073072.
66. Zhong, C.; Xu, H.; Ye, S.; Wang, S.; Li, L.; Zhang, S.; Wang, X. Gibberellic Acid-Stimulated Arabidopsis6 Serves as an Integrator of Gibberellin, Abscisic Acid, and Glucose Signaling during Seed Germination in Arabidopsis. Plant Physiol 2015, 169, 2288-2303, doi:10.1104/pp.15.00858.
67. Yan, A.; Wu, M.; Yan, L.; Hu, R.; Ali, I.; Gan, Y. AtEXP2 is involved in seed germination and abiotic stress response in Arabidopsis. PloS one 2014, 9, e85208, doi:10.1371/journal.pone.0085208.

© 2020 by the authors. Submitted for possible open access publication under the terms and conditions of the Creative Commons Attribution (CC BY) license (http://creativecommons.org/licenses/by/4.0/).
